# Violations of Expectations As Matter for the Believing Process

**DOI:** 10.3389/fpsyg.2017.00772

**Published:** 2017-05-29

**Authors:** Hans-Ferdinand Angel, Rüdiger J. Seitz

**Affiliations:** ^1^Institute of Catechetics and Religious Pedagogics, University of GrazGraz, Austria; ^2^Department of Neurology, Centre for Neurology and Neuropsychiatry, LVR-Klinikum Düsseldorf, Medical Faculty, Heinrich-Heine-Universität DüsseldorfDüsseldorf, Germany

**Keywords:** credition, functional imaging, behavior, valuation, emotion, cognition

## Abstract

For the purpose of this communication it is postulated that violation of expectation means a disturbing event or conflict interfering with a previously established mental state that affords a firm belief or confident feeling. According to this hypothesis a violation of an expectation contradicts predictions and intentions that have been attained on stored experiences, valuations, and actual mood. We will argue that the notion of belief as static or stable which is usually described by expressions such as “my belief” or “our general belief” has to be extended to accommodate the process of belief formation. The credition model emphasizes the procedural aspect of belief by which the “process of believing” becomes similar to other psychological processes. We will describe that the “violation of expectation” can be decoded from the credition perspective and has brain functional correlates.

## Introduction

In this paper we will argue that expectation for something to happen in the future is an important matter for the believing process. Consequently, we understand a violation of expectations as well as a matter for the believing process. More precisely, we want to introduce the idea that believing processes are underlying expectations as well as behavioral and neurophysiological reactions on their violations. To underpin this notion we will show that it is possible to describe reactions on such a (cognitive) conflict within the theoretical framework which is set by the novel model of credition. This approach can enrich the discussion about the violation of expectations by theoretical aspects which have not been discussed so far. Of critical relevance for this discussion is the model of credition which will be explained in some aspects in this contribution. Doing this involves multilevel data mapping (Paloutzian and Park, 2013; Paloutzian and Mukai, 2017) or bi-directionally “translating” the data and concepts from one level of analysis to an adjacent level of analysis in order to assess the degree to which they correspond. Specifically, this paper addresses violation of expectations at both a psychological and a neuroscientific level of analysis. The psychological level describes the mental processes involved in imagining, rendering beliefs out of a complex world of ambiguous information, and in the various verbal and conceptual puzzles created thereby. The neuroscientific level describes research on how these processes work in the human brain.

Thus, we want to discuss (1) a general hypothesis and (2) a specific hypothesis which is based on the general hypothesis.

Hypothesis 1: Violation of expectation involves believing processes.
Hypothesis 2: Within the model of credition “violation of expectation” can be expressed in relation to the so-called enclosure function.

To make understandable that “violation of expectation involves believing processes” we will show that it is possible to express “violation” in terms of credition (parts II–IV). To show that within the model of credition “violation of expectation” can be expressed in relation to the enclosure function we want to work out this aspect by translating a given example into a credition related language (parts V–VII). This intention requires a step by step presentation of the constituting features of the model of credition.

## The Believing Process – a New Perspective on Violations of Expectations

It is uncommon to talk about the believing process. Rather, we are familiar with the use of the expressions “belief” or “faith”. This use of nouns is widespread and predominant. Using nouns is not without effect as it insinuates (at least implicitly) the notion of belief as a state (Churchland and Churchland, 2013). Such stable beliefs have been found to follow a digital code, which is either true or false (Johnson et al., 2015). But, assuming the believing process to refer to mental activity or processes, it is more appropriate to apply the verbal expression “to believe”. What on the first glance may give the impression of mere linguistic styling is, however, on the contrary a not trivial shift of understanding. This approach to the question of belief affords on several levels a change of thinking which can be labeled as “From the question of belief to the question of believing”. Some aspects of this transformation have been explained elsewhere (Angel, 2017). But, for this communication we do not only refer to the novel concept of the believing process. We ground our reflections on “violation of expectation” on our model which seeks to simulate the psychological process of believing. This model will be the guideline for our perspective on the matter of violation of expectation.

The conceptual framework of the believing process and the hereof resulting “model of credition” assume that the believing process is a fundamental brain function that happens many times per day in everyday live (Angel and Seitz, 2016). The *model of the process of believing* includes a number of operational subfunctions that show surprising homology to neurophysiological processes as was described in detail recently. Central to the model is the so-called enclosure function which denotes the self-organizing probabilistic assembly of attributes of a given object or event into a coherent mental representation. These coherent knowledge constructs comprehend formal descriptions of the perceived encounters that can be expressed in terms of objective metrics as well as personal values associated with them as described below. Importantly, people employ these mental constructs for selecting the most appropriate for the subject in a given situation. In other words, perception is converted by the so-called converter function into an intended action which is part of and directed within an entire space of action. This cybernetic model assumes that the mental operations are mediated by a presumed operator in the human brain and can be stabilized by repetitions similarly to a learning process. Attitudes, hormonal states and pharmaceutical agents can modulate these mental operations.

Accordingly, processes of believing link the past sensory experience of a subject with his/her predictions for the future. These predictions correspond to personal expectations having emotional loadings of high subjectivity. The mental representations of the past experience are probabilistic in nature involving the attribution of subjective meaning to the perceptions (Seitz and Angel, 2014). Conversely, based on such probabilistic representations of the past, future acts are generated that are guided by probabilistic predictions of reward and cost to achieve a given goal (Barsalou, 2009; Angel and Seitz, 2016). As people act in their social environment they are constantly confronted with unexpected events and contradictions by others. In other words, humans experience a violation of their expectations all the time. Accordingly, violation of expectation is a frequent event that subjects need to be able to cope with. In its ultimate form a violation can result in a complete negation of an expectation. In this case it will lead to a heavy emotional challenge in the expecting subject influencing his/her subsequent behavior. Thus, there is good reason to assume that humans have to learn to cope with violation of their expectations. Such violations of expectations are defined events, while in contrast the probabilistic representations of meaning making and expectation have evolved over time by repetitive exposure and behavior. Thus, a violation of an expectation can – but does not necessarily – lead to a modification of the probabilistic representation or a certain belief.

In addition, subjects value objects and events in the outside world in terms of personal relevance (Seitz et al., 2009). These value judgments include introspection, goal values, decision values, and prediction errors (Hare et al., 2008). Here, we would like to define valuation as the process by which objects and events are evaluated by acting subjects in terms of utility and benefits. The probabilistic judgment is the default first person perspective of “what does it mean to me?” (Seitz and Angel, 2014). The judgment is loaded implicitly with emotional categories such as happiness, anger, fear, surprise, and disgust. These emotions induce immediate reactions of the subject and typically induce sensations from the inside of the body including raised and strong heartbeat, trembling, and heat in the head as was argued by Damasio (1998). The personal judgment involves automatic emotional processing as well as controlled cognitive processes as shown behaviorally and using event related recordings (Morewedge and Kahneman, 2010; Leuthold et al., 2015). These processes can eventually become consciously accessible to the subject being critical for guiding the subject’s behavior.

Common to these cognitive processes is the relation to subjective categories such as memories, attitudes, desires, and hope (Corlett et al., 2004; Seitz et al., 2016). But these subjective categories can also be abstract categories of general value such as moral, justice, and ethics. Value judgments based on subjective perspective-taking are intimately linked to self-awareness which includes self-esteem, self-other distinctions, and the distinctiveness of one’s own thoughts (Young and Pigott, 1999; Gallagher, 2000). Thereby, people experience themselves as causal agents and authors for their own actions and behavior resulting from a *post hoc* construction of an unconscious decision-making process (Gallagher, 2000; Wegner, 2003). Importantly, subjects judge the credibility of their inferences and predictions in terms of trustworthiness, convincingness, and substantiating evidence. In the positive case the subject arrives at the conviction that he/she accepts this personal interpretation as true or granted and, thus, personally relevant. Consequently, the subject believes it, since or although he/she does not know whether the information is really true (Seitz et al., 2016). Also, the emotional loading is part of the probabilistic mental representation of objects or events determining their relevance for the subject and the expectation the subject has concerning them. Ultimately, this can be translated to the realm of moral and ethics applying in groups and societies (Seitz et al., 2016). Accordingly, a violation of such an expectation is an emotional violation which will heavily affect the given subject’s attitude what to learn already during infantile development (Stahl and Feigenson, 2015). Similarly, extinction learning has been shown to be able to profoundly influence behavioral patterns as in anxiety disorders (Pittig et al., 2016).

## The Believing Process – Implicit Changes of for the New Perspective

To understand “violation of expectation” from the perspective of the believing process we will describe explicitly the underpinnings of this innovative perspective. On the way from the question of belief to the question of believing we are elaborating here three aspects which are fundamental for the transformation of the traditional belief-related thinking.

### Credition: Noun to Verb

It is a huge shift of paradigm to transform the noun-related concepts of “belief” into verb-related concepts. The focus on the topic of the process of believing can be expressed more precisely by the notion “while someone is believing”.

### Credition: Process

The mental activities underlying believing we encompass by the term credition. Importantly, they are to be understood as processes. This raises the question “what is a process”. Here, we touch upon a long history of European thinking which has one of its excellent starting points in an understanding of the world as “fluent” which was brilliantly expressed by 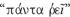 (*panta rhei*, everything flows) as ascribed to the pre-Socratic Heraclitus. Also in modern philosophy there is a vivid discussion about the epistemic state of process thinking. This term was developed as a broad field of interest – it is controversial whether one should speak about process philosophy – and spawned in the writings of Bergson, Merleau-Ponty, and Whitehead, indicating that process constitutes change and occurs through and interacts with time. Time again is a highly controversial concept in philosophy and the understanding of time cannot be reduced to the matter of “measuring” time. We propose that to describe normal believing processes there is a need for a process-theoretical foundation (Angel, 2015). To transform noun-related concepts which understand belief in a static sense into a time-related concept of fluid processes of believing affords to bear on process theoretical concepts. Thereafter, the task of exploring to what extent the structure of credition is compatible with Whitehead’s Metaphysics of Experience may be undertaken (cf. Maaßen, 2017).

### Credition: Not on Religion

Finally, it has to be stated explicitly that the concept of credition is not located in the frame of religion. In fact, we want to stress that credition is not understood as a “religious” process. It is important to mention this as there is often a spontaneous association of religion with “belief/to believe”. This connection between faith and religion has been coined by a long tradition of Western thinking. However, under a procedural perspective this connection is misleading. Importantly, credition applies to religious and secular contexts and it is not a prerequisite to refer to religion in order to understand credition (Seitz et al., 2016).

## Model of Credition – Basic Aspects

Until shortly, there has been no term for the “believing process” that encompasses the notion in common language as well as in philosophy or cognitive science. To address this terminological challenge which hindered the interdisciplinary discourse the term “credition” was introduced into the scientific discussion (Angel, 2013a). The concept of “credition” originated from an anthropological view on religious experiences and consecutively from the attempt to understand “religiosity” (Angel, 2013b). Notably, the neologism “credition” was coined to denote believing processes that encompass both religious and secular beliefs. The term is derived from the Latin “*credere*” (to believe) and is shaped in analogy to other psychological terms like cognition (lat: *cogitare* = to think/to reflect) or emotion (lat. *movere* = to move).

The concept of credition claims that normal believing is inextricably interrelated with cognition and emotion (Sugiura et al., 2015; Angel, 2016). That brings the question on the floor how we can conceive the interaction of credition with interdependent cognitive and emotional processes. The model of credition proposes that believing comprises neuropsychological functions that overlap but do not equal those in cognition and emotion (Angel and Seitz, 2016).

In order to express “violation of expectation” in terms of the credition model it is necessary to outline some basic features of the credition model. It has to be mentioned that for the purpose of this presentation we assume the model of credition as given though there is ongoing scientific research on the character of the believing process^1^. For the reason of this paper the model of credition is sufficiently stable as it is supported by many data of different fields of research. Further, we postulate that violation of expectation means a disturbing event interfering with a previously established mental state that has afforded a firm belief or confident feeling. It should be emphasized that the believing process which has resulted in a firm belief or confident feeling belongs to the past. In contrast, the probabilistic expectation based on the outcome of the believing process which pertains to the future is violated by a momentary event.

### Bab and Bab-Blob-Configuration

In the credition model the hypothesized processes are brought about and act upon meta-theoretical units to which heuristic labels were assigned. For this purpose we describe in the following paragraphs (a) the term “bab” and in consequence derived concepts as there is blob, bab-blob-configuration, and “characteristics of a bab”, and (b) the enclosure function which has been introduced as one of four supposed functions of credition. Notably, one cannot describe the enclosure function without referring to the characteristics of babs. Vice versa, any explanation of any property of the relevant bab or of the property of the bab-blob-configuration is meaningful for an understanding of the enclosure function. The terms “bab” and “blob” are novel and have not existed so far (at least not in the here proposed sense). Why was it necessary to introduce those new terms? Two main reasons are:

The first reason is that recent scientific findings change our view on the relation of emotion and cognition but have not influenced yet our everyday language. “Bab” is a term which reflects these findings. The second reason is that a basic unit for credition is needed and the term (and concept of) “bab” can be offered as such a basic unit.

#### Overlapping Procession of Emotion and Cognition

Emotions and cognitions are considered as two different domains covering separate and partially contradictory aspects of brain function. There is empirical evidence from neuroimaging findings that emotion and cognition are processed in overlapping areas of the lateral prefrontal cortex by which both can contribute to the control of thought and behavior (Gray et al., 2002). Moreover, current data provide converging evidence that working memory and bioelectric activity in lateral prefrontal cortex can be influenced by affective variables (Schaefer and Gray, 2007; Roux et al., 2012). While emotions have been shown to involve the amygdala and the orbitofrontal cortex (Rolls, 2006), cognition comprises different aspects of mental activity such as speech production, memory processes, attention, and learning processes which are processed across widespread circuits in parietal, temporal, and frontal cortical areas as well as in the amygdala (Toga and Mazziotta, 2000; Schaefer and Gray, 2007). Beliefs are important to consider, as they were shown to influence reasoning and brain activity related to reasoning (Goel and Dolan, 2003). A given proposition, therefore, can differ in its personal emotional meaning.

As the European languages do not provide a term to express the overlap of cognition and emotion in a meta-theoretical sense, there is a discrepancy between the capacity of actual language(s) and the actual state of knowledge showing the need to supplement the word pool with terms which can express those given facts. To implement the neuroscientific findings into the frame of linguistic possibilities the term “bab” has been proposed (Angel, 2013a; Angel and Seitz, 2016). The term “bab” indicates in a linguistic, not in a mathematical sense: *“proposition + emotion”.*

#### Bab as Basic Unit of the Believing Process

The model of creditions emphasizes the process character of believing and by this the fluidity of beliefs. One of the most crucial questions is how to define the basic unit of the believing process. It is important that such a unit accommodates two basic claims:

• First, it has to provide a theoretical frame which accounts for the fluidity of the believing process and which allows to integrate different scientific descriptions (physical, biological, neural, behavioral, and so on).
• Second, it has to provide the possibility to integrate cognitive and emotional processes under a common label.

The term “bab” complies with both demands and we propose this term for such a new umbrella-term which has the capacity to indicate the basic unit of credition (see Supplementary Material Box 1).

Having declared “bab” as basic unit we can describe different characteristics which we assign to a single “bab”. The term “blob” is used to indicate a subliminal “bab”. We will come back to the question of subliminal processing below when we discuss the enclosure process.

First, we have to draw the attention to the characteristics of a bab. Owing to its mental function four characteristics can be assigned to a bab – and consequently to every single bab in a bab blob configuration.

• The propositional content: a “bab” can be described as a proposition as for example: “I see something red” or “I fell something sharp”. The proposition becomes explicit by statements such as: “I see this ball to be red” or “I feel this knife to be sharp”.
• The emotional moment: for example, a red light may be perceived as beautiful, warm or attractive, whilst a sharp item may be unpleasant, harmful and, thus, frightening. Note, that the term “bab” comprises the subliminal emotional moment in addition to the propositional content. When this information is expressed verbally, the “bab” will reach explicit awareness both in the speaking and the listening subject.
• The sense of mightiness: the perspective of a subject on a “bab” is not limited to the valence of an emotion but also includes the intensity of the emotion which is reflected by the “sense of mightiness”. Thus, this scaling of an emotion as strong or weak is inherent in the proposition of a “bab”.
• The sense of certainty: this characteristic reflects the conviction of an individual that a “bab” reflects the property of an object or event. The same proposition of a bab can have a high degree of certainty while for others it is uncertain. For instance, “I see something red” or “I see something sharp” has a high degree of certainty in daylight but a low degree of certainty in faint light.

Notably, in a believing process “babs” do not “exist” as single “monades” but as composite “bab-configurations”. Specifically, “babs” include physical attributes such as color and form and personal attributes such as subjective meaning and relevance. In fact, “babs” represent pieces of knowledge with emotional loadings which are assembled into coherent knowledge constructs, the so-called stabilized “bab-blob-configuration” (see Supplementary Material Box 2).

### The Four Functions of Credition

As outlined in the credition model, the believing process consists of four conceptually successive – but nevertheless in reality heavily interwoven – mental functions which are called enclosure function, converter function, stabilizer function, and modulator function (see Supplementary Material Box 3). Notably, one can speak about “converter function” or “converter process” depending on the perspective, which one choses to apply. In the following sections we will explain some aspects of the enclosure function.

With regard to the limitation of space we do not discuss more extensively the other functions in this paper. Just to mention that the converter function means that perception is converted into an intended action which is part of and directed within a space of action. This process employs the prediction of cost and reward and the expectation of future events inherent in a belief (Angel and Seitz, 2016). This cybernetic model of credition assumes that the mental operations are mediated by a presumed operator in the human brain and can be stabilized by repetitions similarly to a learning process. Attitudes, hormonal states, pharmaceutical agents and physical threatening that act on the entire individual can severely influence or modulate these mental operations.

We will not discuss the stabilizer function which is relevant for integration of experiences and their integration into a broader balance-dependent meaning making structure. What we want to state is that these three functions are regarded as universally effective functions whereas the fourth function which is called modulator function is strictly bound to individuals.

### The Enclosure Function

**FIGURE 1:**
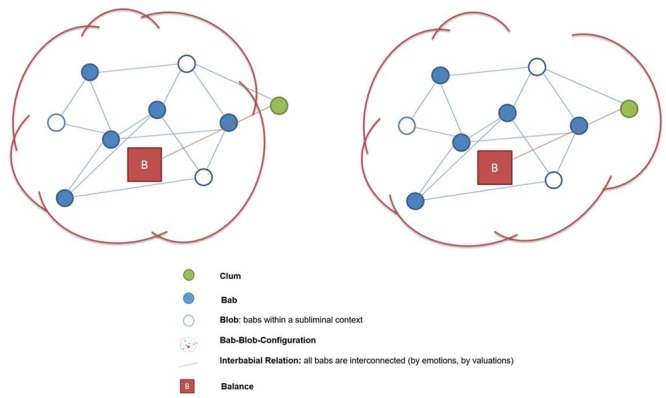
**The enclosure function involves the composition of identified (bab) and subliminal (blob) properties of an object or event.** These compositions are balanced but in continuous evolution allowing the enclosure even of a property with opposite valence (clum).

In addition to neuropsychological topics such as perception, action, valuation, and stabilization one of the subfunctions of the model of credition is the so-called enclosure function. It denotes the self-organizing probabilistic assembly of mental attributes. Thus, the enclosure function is a mental process constituting or modifying “bab-configurations” or – in other words – different features of an object or event which are linked to each other to determine their characteristics and value. Under this perspective bab-configurations are subsets of mind-sets which are activated when a process of believing starts (Angel, 2013a; Angel and Seitz, 2016). The coherent knowledge constructs comprehend formal descriptions of the perceived encounters that can be expressed in terms of objective metrics as well as personal values associated with them. The personal values reflect the meaning and relevance the object or event has for the given individual (Seitz and Angel, 2014). Note, that the psychological description of the mental processes involved in imagining, making beliefs out of a complex world of ambiguous information, and of the various verbal and conceptual puzzles created thereby goes beyond the topic of this paper. Therefore it is reasonable to assume a systems level which is composed of a number of different meaning making processes and allows for flexible rearrangements of different meanings over time (**Figure 1**).

As many stimuli do not reach our consciousness, we have to accommodate also the subliminal aspect (Teske, 2007) in the credition model. As mentioned, for a bab which remains subconsciously the artificial term “blob” was introduced. That is the reason why we should speak of a “bab-blob-configuration” rather than of a “bab-configuration”. We suggest that effects of placebo or nocebo (Myers et al., 1987; Benedetti et al., 2006; Jensen et al., 2012) are prominent examples for accounting for such a believing process (Meissner, 2017).

The term “clum” indicates the irritating moment which is in debate during the enclosure process. The name enclosure process is derived from the function by which an irritating clum is “included” or not into a bab-blob-configuration. The inner process which takes part in the period of “open result” comes to its end when the clum will be integrated or not in a previously existing bab-blob-configuration. Among other aspects processes of valuation are influential. Therefore, the enclosure function is interconnected with processes of valuation.

The enclosure process challenges the so-far existing bab-blob-configuration. In course of this process previously acquired “knowledge” which is stored in the actual bab-blob-configuration will be adjusted according to novel external stimuli and inner value terms associated with them. This process of adjustment is related to the inner balance system as well as to the meaning-making system. The believing process serves to cope with homeostatic challenges. On a basic level we can see homeostatic bodily processes. Finally, we have to stress that a clum also is a “bab”, but one with a specific property during the enclosure process.

## “Violation of Expectation” in Terms of Credition

Based on the model of credition a “violation of an expectation” can be understood as a mental process which leads to the “*realization that a given bab-blob-configuration includes (or included) an inadequate bab.*” Within the framework of the credition model the specific characteristic of a “violation of expectation” is related to the so called “clum” which indicates an irritating moment. A “clum” has a crucial relevance for the so-called enclosure function and by this for the initiation of a process of credition. But with regard to an expectation a “clum” must have a well-defined property. According to our understanding that “violation of an expectation” can be defined as “realization that a given bab-blob-configuration includes (or included) an inadequate bab” we can formulate the hypothesis: *the propositional content of a clum is identical with one of the babs in the agent‘s configuration but (mathematically spoken) with a negative algebraic sign.*

As an example for a “violation of expectation” in terms of credition we present the following situation. The example is that someone has booked a flight. Accordingly, the person believes that he/she will be in the position to travel to the desired destination and has engaged in the actions mandatory to prepare this trip. When approaching the gate the person expects to receive the boarding pass and to get on the plane. But then the person is confronted with the unpredicted information “the flight is overbooked”.

Our following discussion refers mainly to the *characteristics of babs* as well as to the *enclosure function* of credition. Nevertheless, we want to draw the attention to the fact that our given explanation is not a comprehensive description of the enclosure function but will highlight only some of the indispensable aspects.

### Irritation as Production of a Clum

The fact or event which violates an expectation has to be described as “irritating moment” and transformed in such a way that it can become a “clum”. As mentioned, detecting an irritating moment is the normal precondition for any beginning of a believing process and the initiation of an enclosure function (cf. part III). In so far it is a matter of perception if something is detected as irritating signal. In our example the irritating moment “the flight is overbooked” is communicated as information in words and addresses the auditory sensory system. Of course the characters of signals and the mode of their perception can differ heavily. For instance, processing a perceived *static object* differs in several aspects from processing a perceived event which has to be coded temporally. But the differences related to the property of the perceived “irritation” do not change the general explanation of how a clum is integrated.

How can we conceive the above mentioned hypothesis that the propositional content of a clum is identical with one of the babs in the agent‘s configuration but (mathematically spoken) with a negative algebraic sign? For answering this question we have to explain what aspects can be ascribed to a clum in case of “violation of expectation”. For this we have to clarify what might be the propositionally identical content of a clum and of a bab. Here, we have to acknowledge that the notion of “violation” can only be understood as a distinct event in time, while a belief pertains over time. This means that the concepts of believing and of violation accommodate different temporal aspects.

To understand the “character of the violation” we have to start at the moment when the “frame” for a possible violation was settled. In our example this is the moment when the booking of the flight was accomplished. After having booked the flight a person will have established a mental state that affords a firm belief or confident feeling that he or she will be able to use exactly this flight. We can translate the end of the booking operation like follows: the agent has included into his or her bab configuration a bab with the propositional content “the flight is available”.

### Connection of Cognition and Emotion

As a “bab” by definition is understood as “proposition plus emotion” (cf. part III) we assume that the emotional loading of this specific “bab” will be “joy”. We cannot discuss here how the emotional loading (joy) interacts with the cognitive process which takes as rationally undoubtable that the “flight will be available”.

### Interbabial Relations

Nor can we discuss how the emotionally positive bab “flight will be available” interacts with other babs in the configuration. Of course, these configurations will differ for different subjects depending on their individual experiences. If someone never has come into such a situation he or she probably will not have included an emotionally mighty bab “flights are not guaranteed by booking”. On the contrary, a frequent flyer will have integrated such a bab in his or her configuration.

### Propositional Contradiction of the Clum and One of the Babs

Now imagine what will happen when the person gets the information: “the flight is overbooked”. In order to be able to verify the hypothesis we have to check whether this information can be translated into a formulation which is identical with the propositional content of the bab “the flight is available”. Under linguistic aspects the information “the flight is overbooked” is negatively identical with the propositional content of the bab “the flight is available”. Thus, after getting the overbooking information we have the following situation:

• Bab in the bab configuration:
“the flight is available” plus emotion “joy”
[ (+ proposition) × emotion(1) ]
• Clum
“the flight is not available” plus emotion “anger”
[ (- proposition) × emotion(2) ].

As mentioned this formulation is understood linguistically, not mathematically. Mathematically, it should be written as product because the emotion does not come additionally to the proposition but simultaneously. Thus, the use of the term “bab” stresses this interconnectedness of propositional and emotional aspects. When the person “believes” that the flight is overbooked he or she has to integrate the clum into his previously established mental state. After the integration of this negatively loaded clum also the emotional value of his or her bab-blob-configuration will have been changed into a more negative set. Besides, the full integration of the clum into the bab-blob-configuration marks the end of the enclosure process.

### Bab and Clum: Cognitive Dissonance

When regarding the content level we will observe a mental dynamic which is caused by the interaction of two contradicting babs. This kind of problem is described by the concept of cognitive dissonance (Festinger, 1957). In his influential cognitive dissonance theory, Festinger included believing in the class of dissonance reduction processes. Accordingly, believing is to change or to add a cognitive element to reduce dissonance with or between other cognitive elements. For example, the dissonance between two ideas, a belief that people are good in general, and a knowledge that children go through a period of aggressive behavior, is reduced by believing existence of malevolent ghosts which enter into children and cause them to do inappropriate things. The idea of dissonance reduction appears to fit well with the explanation of human brain function in the free-energy principle as an optimizing machinery for value and its counterpart surprise (Friston, 2010). Fundamental herein are the probabilistic predictions of value or reward concerning perceived information and of expected error or cost concerning future actions, which drive the system to the next state by a simple principle of reducing the free energy. Believing is one of the conscious expressions of such a self-organizing process.

### Bab and Clum: The Degree of Certainty

The degree of certainty of the bab “the flight is overbooked” may differ according to experience. Though everybody knows theoretically that “flights are never guaranteed by booking” an agent may act during further steps of decision making as if the bab in question has a high degree of certainty and not prepare a plan B while another agent may attribute a lower degree of certainty. In everyday language he or she might comment “one never knows”. In terms of credition the degree of certainty influences the activity among the babs within the bab-blob-configuration. A lower degree of certainty will have as consequence a more fluid configuration which results in a higher flexibility of the agent.

### Bab and Clum: Mismatch of Emotions

On the level of emotions we will have turbulences which are caused by the interference of two distinguished emotions – joy and anger. That brings up the question what happens with a bab whose propositional content has a double-loading with different emotion. How will the originally emotional loading “joy” be infected by an arising anger? Will the anger be raised due to the original joyful base or will it be generated spontaneously without recall of the original joyful state? Questions like these open the field for discussions of emotional interaction. Taking into account a “circumplex model of emotions” one can develop a differentiated view on emotions and assume that different emotions influence each other. One can discriminate primary and secondary emotions and assume families of emotions based on similarity (Plutchik, 2001).

### Bab and Clum: The Mightiness of Emotions

Partly the mightiness of the emotional loading of the clum “flight is overbooked” will depend on the alternatives. If someone deplores to miss a marvelous concert due to the early flight the information that the flight is overbooked might stimulate as first reaction that there will be a chance now to visit the concert.

### Bab and Clum: Match of Propositional Information and Emotions

But, from the perspective of credition the focus of interest will be on the question: how the turbulences can be described which are caused by interference of emotion and information. Here the question is to be discussed whether and in which way emotions can be seen as information (Schwarz, 2001, 2011). Of course, the enclosure process is a question of energy. Partly, it is energy consuming and has to be observed under respect of free energy, partly it will set free energy which can be used for action (Friston, 2010).

### Enclosure Function and Time for Integration the Clum

Another aspect is the question of time. How long does it take until the clum “the flight is not available” is incorporated? That is identical with the question of how long the enclosure process will take place. On a fundamental neurophysiological level this is an open question.

However, an important and unanswered question comes to the surface: is what constitutes the knowledge that is stored in the brain merely deposited at once as facts and information, or is it the result of processes (Krüger et al., 2009)? Strong arguments have been made for both views. Experiments on brain–computer interfaces provide good evidence that processes are among the things represented in the brain because, e.g., subjects can learn to actively modulate their brain activity in order to move their paralyzed arm or to write words and even sentences (Birbaumer, 2014).

### Further Aspects

The model of credition provides a couple of further aspects which should be taken into account when describing the character of the possible interaction of a clum with a bab-blob-configuration. This would be for instance the influence of subliminal processes on the consciously perception of a violation. We suppose that these subliminal effects which can be described on neuropharmacological (Holzer, 2017) and microbiological (Sensen and Berg, 2017) levels have to be taken into account in a much broader sense than we have been used to acknowledge. Or, to give a second example, the role of the characteristics of babs should be discussed more deeply with the violation process. This would give deeper insights into the effects which result from a change of emotional mightiness (from mega-bab to mini-bab or inverse). In a similar manner it should be reflected how a modification of the degree of certainty has to be understood – as a sudden event or as an act which is going to happen in a creeping way (Huber and Schmidt-Petri, 2009). Or, again another point: a broader discussion would be needed about how we can understand the interaction of the mere biological homeostatic balance system with the higher level (quasi-homeostatic) system of meaning-making. But those aspects we have to omit with regard to space.

## Hidden Polyvalence of the Notion “Violation of Expectation”

It might be trend-setting to identify believing as a crucial process which influences the development of expectations as well as the handling of their violations. This will allow us to conceive “expectation” as a (preliminary) stabilized state resulting from continuously running believing processes. “Violation of expectation” can be interpreted as an event which reopens the next turn of believing processes that end with the final integration of the violating clum into the reorganized bab-blob-configuration. Using the perspective of the model of credition we can state that the expression “violation of expectation” is an umbrella term which covers a wide range of possible notions. The model of credition allows us to understand the semantic ambiguity which is inherent in the notion “violation of expectation”. We will explain this view with a few examples of decoding possibilities afforded by the credition model.

*“Violation of expectation”* can be decoded as:

### Change of the Emotional Shape of a Bab Configuration after Integration of the Clum

In our example the clum “flight overbooked” probably will be combined with negative emotion like anger. When the enclosure process comes to its end the negatively shaped proposition “flight overbooked” will be integrated into the former bab-blob-configuration. This of course will influence the emotional shape of the entire bab-blob-configuration and it can be observed how the emotional loading of the clum will influence in course of the time the configuration. This process can be conceived as a coping process. On a psychological level we will find as a result the modified mood of the person.

### Obscuring the Space of Action and Hindering Decision Making

One can interpret the integration of the clum with regard of the converter process (which is relevant for the configuration of the space of action). In this case is relevant that an integrated clum will destabilize the existing bab-blob-configuration. As a result we will see a modification of the impulses which are relevant for action. It will be less clear “in which direction” the space of action will be opened. As the space of action is understood as the preliminary state of a decision, the ambiguity of impulses can be understood as an obstacle for a quick decision.

### Destabilization of the Balance System

In case of great importance of the previous integration of the bab “flight available” the clum “flight not available” can have strong consequences. Depending on the emotional mightiness of the clum the integration can touch heavily on the balance system. We can easily imagine the case that the flight was booked to visit a beloved person of poor health. The need to integrate the clum “flight not available” might touch the traveler’s balance system and provoke serious bodily reactions.

### Reopening of the Believing Process

In the case that the destabilization of bab-blob-configuration is detected and perceived as an irritation a next turn of the believing process can commence. This does not predict in which direction the space of action will be opened. It will definitely be different when the now upcoming clum has the proposition “change needed” or the proposition “not with me!”

Finally, we want to mention that it will be possible to interpret standard positions toward “violation of expectation” in the light of the model of credition. Using the language of credition it will be possible to assign the concept of the believing process to existing models of expectation. For instance Bandura’s concept of self-efficacy can be interpreted in terms of credition as “existence of a so-called mega-bab with the properties: [a] the proposition/content “I am efficient”, [b] positive/joyful [c] emotional loading by which the [d] degree of certainty of the proposition is augmented. When trying to translate Pavlov’s concept of conditioned reflexes into a language of credition we would focus more on the relation of the modulator function and the stabilizer function.

## Neurophysiological Foundation of Processes of Believing and Their Violation

Functional magnetic resonance imaging (MRI) is suited to identify the areas involved in the working human brain. As we have outlined above, the believing process is an integrative brain function involving a number of psychophysical subfunctions. Here, we are outlining some recent empirical data about the implementation of such integrative functions in the human brain. Most information is in the subliminal or preconscious domain but, nevertheless involves the activation of extensive cerebral networks including the lateral prefrontal cortex (Changeux and Dehaene, 2008). In particular, gamma-oscillations have been advanced as a candidate functional expression for binding information of different origins into a coherent representation in working memory (Roux et al., 2012). The global workspace integrating perception and valuation and allowing for generation of appropriate action is critically modified by previous experience and by the momentary focus of attention (Koechlin and Summerfield, 2007; Mesulam, 2008; Dehaene and Changeux, 2011). In this process identification of conflict – the violation of expectation – is of fundamental importance. From a large body of evidence in the open literature we know that the anterior cingulate is a critical node in processing conflict (Carter and van Veen, 2007). A further important field of interest with relevance to the discussion in this paper is the generation and inhibition of behavior. This is due to the fact that a violation of expectation influences the individuals’ behavior by affecting their prospects of long- and short-term reward. MRI studies showed that normal preparatory activity in the premotor and posterior parietal cortex can be modulated by the subjective absolute value (in terms of monetary consequences) of an upcoming action (Iver et al., 2010). Specifically, subjects who had large gains and believed they performed well, and subjects who had large losses and believed they performed poorly, had the highest preparatory signals. The neural activity in the medial frontal gyrus appears to link unexpected sensory information including violation of reward prediction (Martin et al., 2009; Schwartenbeck et al., 2016) with preparatory control of arm movements but also affording response inhibition and task switching (Rushworth et al., 2002; Leung and Cai, 2007; Chen et al., 2010). In particular, the supplementary motor area (SMA) was shown to be involved in free choice movement coding (Nachev et al., 2008; Passingham et al., 2010; Pfurtscheller et al., 2014). The SMA and premotor areas are also involved in judgment of aesthetics as well as brightness, which signifies that the SMA has more general behavioral relevance (Ishizu and Zeki, 2011, 2013). Conversely, a number of distinct nodes in the medial frontal cortex, including the SMA and pre-SMA, are involved in the proactive and inhibitory control of actions (Seitz et al., 2006; Van Overwalle, 2009).

In addition to cortical brain areas, such an integration of this different type of information was shown to take place by involvement of the basal ganglia. There is evidence from rat T-maze experiments that activities modulated to different frequencies can develop in parallel in different subregions of the striatum, allowing for a coordinated flow of information through different *trans*-striatal networks and, thereby, for simultaneous and independent operations in separate networks (Thorn and Graybiel, 2014). Furthermore, the modulation of cortical information by processing in *trans*-striatal relay loops has been described as of key importance for learning routines and rules as well as their combinations (Graybiel and Grafton, 2015). Recently, it was shown that shifts in beliefs involve dopamine-rich midbrain regions (Schwartenbeck et al., 2016).

Since the individuals’ capacity to deal with on-line information is limited (Baddeley, 1981), probabilistic representations and predictions assist the person to arrive at behavioral decisions. This is because beliefs can be envisioned to guide the individual’s choices, although they limit his/her space of action. MRI studies showed that preparatory activity in the premotor and posterior parietal cortex is modulated by the subjective absolute value of an upcoming action (Iver et al., 2010). A compelling argument for the relevance of functional neuroanatomic data comes from neurological patients showing that a given neuropsychic function is impaired due to damage to a certain brain structure that is involved in executing this function in healthy volunteers. A large meta-analysis of 193 studies showed that a loss of gray matter in brain structures belonging to the salience network, including the anterior insula and dorsal anterior cingulate, was related to deficits in executive functions in patients with different mental illnesses (Goodkind et al., 2015).

Studies of this sort show that the brain structures mediating adequate behavior in healthy subjects are compromised in mental illnesses. Although there is no causal link, it is likely that the integrative brain functions such a meaning making, prediction of future events, control of behavior and realizing of a violation of expectation are impaired in such patients. For example, patients with delusions have severe deficits in mental processing of perception, memory, bodily agency, social learning and are, thus also impaired in predicting future events in the external world (Corlett et al., 2010). Likewise, neuroimaging studies in psychopaths have shown that these persons are impaired in increasing activity in the anterior insula (Sitaram et al., 2014) which was paralleled by lower conditioned fear responses (Veit et al., 2013). In addition, the so-called alien limb syndrome which represents a violation of the sense of body integrity has been related to damage of the parietal cortex (Graff-Radford et al., 2013) and the medial frontal cortex (Feinberg et al., 1992; Biran et al., 2006) the latter of which was also related to an abolished self-reference (Philippi et al., 2012). Evidence from functional imaging studies has revealed that the medial fronto-parietal circuit is critically abnormal in post-traumatic distress disorder reflecting altered mental functioning secondarily to a profound violation of the sense of safety (Cwik et al., 2014). In fact, important aspects of believing, such as personal reference, empathy, and adequate control of behavior, appear to rely on the integrity of the medial and lateral prefrontal cortex. Adequate control of behavior means resistance to react in case of violation of expectation, which is possible even with a low time limit of the cueing and/or go-signal of about 200 ms (Schultze-Kraft et al., 2016).

### Limitations

By this paper we hope to contribute to a more comprehensive understanding of the complex interaction of violation of expectation and the process of believing. This can be interpreted as a severe conflict of prediction error with previous experience. To the best of our knowledge we do not know of any other model of the believing process. We would like to open a new field of discussion as beliefs and the believing process appear as “possible targets for neuroscientific research” (Seitz, 2017). Our discussion here reflects mainly the question of how and to which extent previous and current, in principle, static approaches to the question of belief can contribute to our understanding of the *process* character of belief formation.

There are, however, limitations which are caused by the need to present the believing process and the functions of credition in a condensed manner. A less abbreviated presentation could and should explain many aspects much more in detail.

First, we did not discuss here the whole range of possible aspects. Thus, we omitted for instance the developmental aspect which should be reflected for children, aging persons, and so on. We did not discuss aspects concerning the impacts of traumas on violations of expectation, or more generally the topic of coping as for instance “learned helplessness” (Abramson et al., 1978). Nor did we expand on violation of expectation under the perspective of neuro- or psychopathy, which may be caused by a disturbance of balance (Devinsky, 2009). Moreover, we did not extend the reflection toward other cultural areas.

There are also theoretical limitations which depend on the actual state of research and the available neurophysiological data. There are general limitations which are partly connected with philosophical presumptions. In this regard there are specific limitations which depend on the hermeneutic question of translation the model of credition into an everyday language as well as into a scientifically adequate expression. These limitations may challenge young researchers of different disciplines like philosophy (epistemology, philosophy of mind), psychology, neurology, or with interest in different relevant fields like conflict solving, leadership, or mediation.

## Author Contributions

All authors listed, have made substantial, direct and intellectual contribution to the work, and approved it for publication.

## Conflict of Interest Statement

The authors declare that the research was conducted in the absence of any commercial or financial relationships that could be construed as a potential conflict of interest.

## Footnotes

1 http://credition.uni-graz.at/de/credition-basic-research/

## References

[B1] Abramson L. Y. Seligman M. E. P. Teasdale J. D. (1978). Learned helplessness in humans: critique and reformulation. *J. Abnorm. Psychol.* 87 49–74. 10.1037/0021-843X.87.1.49 649856

[B2] Angel H. F. (2013a). “Credition”, in *Encyclopedia of Sciences and Religion* eds Runehov A. L. C. Oviedo L. Azari N. P. (Dordrecht: Springer) 536–539. 10.1007/978-1-4020-8265-8_1565

[B3] Angel H. F. (2013b). “Religiosity”, in *Encyclopedia of Sciences and Religion* eds Runehov A. L. C. Oviedo L. Azari N. P. (Dordrecht: Springer) 2012–2014. 10.1007/978-1-4020-8265-8_1503

[B4] Angel H. F. (2015). “Process and creditions: How to understand the process of believing?”, in *Advances in Process Thought: Society, Education, and God* eds Dziadkowiec J. Lamza L. (Newcastle upon Tyne: Cambridge Scholars Publishing) 265–280.

[B5] Angel H. F. (2016). No believing without emotion: the overlapping of emotion and cognition in the model of credition. *Stud. Sci. Theol.* 15 215–222.

[B6] Angel H. F. (2017). “Credition: from the question of belief to the question of believing”, in *Process of Believing: The Acquisition, Maintenance, and Change in Creditions* eds Angel H. F. Oviedo L. Paloutzian R. F. Runehov A. L. C. Seitz R. J. (Dordrecht: Springer) 17–36. 10.1007/978-3-319-50924-2_2

[B7] Angel H. F. Seitz R. J. (2016). Processes of believing as fundamental brain function: the concept of credition. *SFU Res. Bull.* 1 1–20. 10.15135/16.4.1.1-20

[B8] Baddeley A. (1981). The concept of working memory: a view of its current state and probable future development. *Cognition* 10 17–23. 10.1016/0010-0277(81)90020-2 7198533

[B9] Barsalou L. W. (2009). Simulation, situated conceptualization, and prediction. *Philos. Trans. R. Soc. B Biol. Sci.* 364 1281–1289. 10.1098/rstb.2008.0319 PMC2666716 19528009

[B10] Benedetti F. Amanzio M. Vighetti S. Asteggiano G. (2006). The biochemical and neuroendocrine bases of the hyperalgesic nocebo effect. *J. Neurosci.* 26 12014–12022. 10.1523/JNEUROSCI.2947-06.2006 17108175 PMC6674855

[B11] Biran I. Giovannetti T. Buxbaum L. Chatteriee A. (2006). The alien hand syndrome: What makes the alien hand alien? *Cogn. Neuropsychol.* 23 563–582. 10.1080/02643290500180282 21049344

[B12] Birbaumer N. (2014). Neural signatures of modified memories. *Neuron* 81 3–5. 10.1016/j.neuron.2013.12.019 24411726

[B13] Carter C. S. van Veen V. (2007). Anterior cingulate cortex and conflict detection: an update of theory and data. *Cogn. Affect. Behav. Neurosci.* 7 367–379. 10.3758/CABN.7.4.367 18189010

[B14] Changeux J.-P. Dehaene S. (2008). “The neuronal workspace model: conscious processing and learning”, in *Learning Theory and Behavior* Vol. 1 eds Changeux J.-P. Menzel R. (Oxford: Elsevier) 729–757.

[B15] Chen X. Scango K. W. Stuphorn V. (2010). Supplementary motor area exerts proactive and reactive control of arm movements. *J. Neurosci.* 30 14657–14675. 10.1523/JNEUROSCI.2669-10.2010 21048123 PMC2990193

[B16] Churchland P. S. Churchland P. M. (2013). “What are beliefs?”, in *The Neural Basis of Human Belief Systems* eds Krueger F. Grafman J. (Hove: Psychology Press) 1–17.

[B17] Corlett P. R. Aitken M. R. Dickinson A. Shanks D. R. Honey G. D. Honey R. A. (2004). Prediction error during retrospective revaluation of causal associations in humans: fMRI evidence in favor of an associative model of learning. *Neuron* 44 877–888. 10.1016/j.neuron.2004.11.022 15572117

[B18] Corlett P. R. Taylor J. R. Wang X. J. Fletcher P. C. Krystal J. H. (2010). Toward a neurobiology of delusions. *Prog. Neurobiol.* 92 345–369. 10.1016/j.pneurobio.2010.06.007 20558235 PMC3676875

[B19] Cwik J. C. Sartory G. Schürholt B. Knuppertz H. Seitz R. J. (2014). Posterior midline activation during symptom provocation in acute stress disorder: an fMRI study. *Front. Psychiatry* 5:49 10.3389/fpsyt.2014.00049 PMC4021128 24847285

[B20] Damasio A. R. (1998). Emotion in the perspective of an integrated nervous system. *Brain Res. Rev.* 26 83–86. 10.1016/S0165-0173(97)00064-7 9651488

[B21] Dehaene S. Changeux J.-P. (2011). Experimental and theoretical approaches to conscious processing. *Neuron* 70 200–227. 10.1016/j.neuron.2011.03.018 21521609

[B22] Devinsky O. (2009). Delusional misidentifications and duplications Right brain lesions, left brain delusions. *Neurology* 72 80–87. 10.1212/01.wnl.0000338625.47892.74 19122035

[B23] Feinberg T. E. Schindler R. J. Flanagan N. G. Haber L. D. (1992). Two alien hand syndromes. *Neurology* 42 19–24. 10.1212/WNL.42.1.19 1734302

[B24] Festinger L. A. (1957). *Theory of Cognitive Dissonance.* Stanford, CA: Stanford University Press.

[B25] Friston K. (2010). The free-energy principle: A unified brain theory? *Nat. Rev. Neurosci.* 11 127–138. 10.1038/Nrn2787 20068583

[B26] Gallagher S. (2000). Philosophical conceptions of the self: implications for cognitive science. *Trends Cogn. Sci.* 4 14–21. 10.1016/S1364-6613(99)01417-5 10637618

[B27] Goel V. Dolan R. J. (2003). Explaining modulation of reasoning by belief. *Cognition* 87 B11–B22. 10.1016/S0010-0277(02)00185-3 12499108

[B28] Goodkind M. Eickhoff S. B. Oathes D. J. Jiang Y. Chang A. Jones-Hagata L. B. (2015). Identification of a common neurobiological substrate for mental illness. *JAMA Psychiatry* 71 1222–1230. 10.1001/jamapsychiatry.2014.1100 PMC4791058 25651064

[B29] Graff-Radford J. Rubin M. N. Jones D. T. Aksamit A. J. Ahlskog J. E. Knopman D. S. (2013). The alien limb phenomenon. *J. Neurol.* 260 1880–1888. 10.1007/s00415-013-6898-y 23572346 PMC3914666

[B30] Gray J. R. Braver T. S. Raichle M. E. (2002). Integration of emotion and cognition in the lateral prefrontal cortex. *Proc. Natl. Acad. Sci. U.S.A.* 99 4115–4120. 10.1073/pnas.062381899 11904454 PMC122657

[B31] Graybiel A. M. Grafton S. T. (2015). The striatum: where skills and habits meet. *Cold Spring Harb. Perspect. Biol.* 7:a021691 10.1101/cshperspect.a021691 PMC4526748 26238359

[B32] Hare T. A. O’Doherty J. Camerer C. F. Schultz W. Rangel A. (2008). Dissociation the role of the orbitofrontal cortex and the striatum in the computation of goal values and prediction error. *J. Neurosci.* 28 5623–5630. 10.1523/JNEUROSCI.1309-08.2008 18509023 PMC6670807

[B33] Holzer P. (2017). “Interoception and gut feelings: unconscious body signals’ impact on brain function, behavior, and belief processes”, in *Process of Believing: The Acquisition, Maintenance, and Change in Creditions* eds Angel H. F. Oviedo L. Paloutzian R. F. Runehov A. L. C. Seitz R. J. (Dordrecht: Springer) 435–442. 10.1007/978-3-319-50924-2_31

[B34] Huber F. Schmidt-Petri C. (2009). *Degrees of Belief.* Heidelberg: Springer 10.1007/978-1-4020-9198-8

[B35] Ishizu T. Zeki S. (2011). Toward a brain-based theory of beauty. *PLoS ONE* 6:e21852 10.1371/journal.pone.002/1852 PMC3130765 21755004

[B36] Ishizu T. Zeki S. (2013). The brain’s specialized systems for aesthetic and perceptual judgment. *Eur. J. Neurosci.* 37 1413–1420. 10.1111/ejn.12135 23373763 PMC3792471

[B37] Iver A. Lindner A. Kagan I. Andersen R. A. (2010). Motor preparatory activity in posterior parietal cortex is modulated by subjective absolute value. *PLoS Biol.* 8:e1000444 10.137/journal.pbio.1000444 PMC2914636 20689802

[B38] Jensen K. B. Kaptchuk T. J. Kirsch I. Raicek J. Lindstrom K. M. Berna C. (2012). Nonconscious activation of placebo and nocebo pain responses. *Proc. Natl. Acad. Sci. U.S.A.* 109 15959–15964. 10.1073/pnas.1202056109 23019380 PMC3465419

[B39] Johnson S. G. B. Merchant T. Keil F. C. (2015). “Predictions from uncertain beliefs”, in *Proceedings of the 37th Annual Conference of the Cognitive Science Society* eds Noelle D. C. Dale R. Warlaumont A. S. Yoshimi J. Matlock T. Jennings C. D. (Austin, TX: Cognitive Science Society) 1003–1008.

[B40] Koechlin E. Summerfield C. (2007). An information theoretical approach to prefrontal executive function. *Trends Cogn. Sci.* 11 229–235. 10.1016/j.tics.2007.04.005 17475536

[B41] Krüger F. Barbey A. K. Grafman J. (2009). The medial prefrontal cortex mediates social event knowledge. *Trends Cogn. Sci.* 13 103–109. 10.1016/j.tics.2008.12.005 19223228

[B42] Leung H. C. Cai W. (2007). Common and differential ventrolateral prefrontal activity during inhibition of hand and eye movements. *J. Neurosci.* 7 9893–9900. 10.1523/JNEUROSCI.2837-07.2007 PMC6672638 17855604

[B43] Leuthold H. Kunkel A. Mackenzie I. G. Filik R. (2015). Online processing of moral transgressions: ERP evidence for spontaneous evaluation. *Soc. Cogn. Affect. Neurosci.* 10 1021–1029. 10.1093/scan/nsu151 25556210 PMC4526472

[B44] Maaßen H. (2017). “The structure of credition in whitehead’s metaphysics of experience”, in *Process of Believing: The Acquisition, Maintenance, and Change in Creditions* eds Angel H. F. Oviedo L. Paloutzian R. F. Runehov A. L. C. Seitz R. J. (Dordrecht: Springer) 217–236. 10.1007/978-3-319-50924-2_16

[B45] Martin L. E. Potts G. F. Burton P. C. Montague P. R. (2009). Electrophysiological and hemodynamic responses to reward prediction violation. *Neuroreport* 20 1140–1143. 10.1097/WNR.0b013e32832f0dca 19690501 PMC4095766

[B46] Meissner K. (2017). “Believing in the effectiveness of treatment: from placebo to credition and back”, in *Process of Believing: The Acquisition, Maintenance, and Change in Creditions* eds Angel H. F. Oviedo L. Paloutzian R. F. Runehov A. L. C. Seitz R. J. (Dordrecht: Springer) 125–137. 10.1007/978-3-319-50924-2_9

[B47] Mesulam M. (2008). Representation, inference, and transcendent encoding in neurocognitive networks of the human brain. *Ann. Neurol.* 64 367–378. 10.1002/ana.21534 18991346

[B48] Morewedge C. K. Kahneman D. (2010). Associative processes in intuitive judgment. *Trends Cogn. Sci.* 14 435–440. 10.1016/j.tics.2010.07.004 20696611 PMC5378157

[B49] Myers M. G. Cairns J. A. Singer J. (1987). The consent form as a possible cause of side effects. *Clin. Pharmacol. Ther.* 42 250–253. 10.1038/clpt.1987.142 3621780

[B50] Nachev P. Kennard C. Husain M. (2008). Functional role of the supplementary and pre-supplementary motor areas. *Nat. Rev. Neurosci.* 9 856–869. 10.1038/nrn2478 18843271

[B51] Paloutzian R. F. Mukai K. J. (2017). “Believing, remembering, and imagining: the roots and fruits of meanings made and remade”, in *Process of Believing: The Acquisition, Maintenance, and Change in Creditions* eds Angel H.-F. Oviedo L. Paloutzian R. F. Runehov A. L. C. Seitz R. J. (Dordrecht: Springer) 39–49.

[B52] Paloutzian R. F. Park C. L. (2013). “Directions for the future of psychology of religion and spirituality: research advances in methodology and meaning systems”, in *Handbook of the Psychology of Religion and Spirituality* 2nd Edn eds Paloutzian R. F. Park C. L. (New York City, NY: Guilford Press) 651–665.

[B53] Passingham R. E. Bengtsson S. L. Lau H. C. (2010). Medial frontal cortex: from self-generated action to reflection on one’s own performance. *Trends Cogn. Sci.* 14 16–21. 10.1016/j.tics.2009.11.001 19969501 PMC2806969

[B54] Pfurtscheller G. Andrade A. Koschutnig K. Brunner C. Lopes da Silva F. (2014). Initiation of voluntary movements at free will and ongoing 0.1 Hz BOLD oscillations in the insula – a pilot study. *Front. Integr. Neurosci.* 8:93 10.3389/fnint.2014.00093 PMC4259110 25538577

[B55] Philippi C. L. Duff M. C. Denburg N. L. Tranel D. Rudrauf D. (2012). Medial PFC damage abolishes the self-reference effect. *J. Cogn. Neurosci.* 24 475–481. 10.1162/jocn_a_00138 21942762 PMC3297026

[B56] Pittig A. van den Berg L. Vervliet B. (2016). The key role of extinction learning in anxiety disorders: behavioral strategies to enhance exposure-based treatments. *Curr. Opin. Psychiatry* 29 39–47. 10.1097/YCO.0000000000000220 26575298

[B57] Plutchik R. (2001). The nature of emotions. *Am. Sci.* 89 344–350. 10.1511/2001.4.344

[B58] Rolls E. T. (2006). Brain mechanisms underlying flavour and appetite. *Philos. Trans. R. Soc. Lond. B Biol. Sci.* 361 1123–1136. 10.1098/rstb.2006.1852 16815796 PMC1642694

[B59] Roux F. Wibral M. Mohr H. M. Singer W. Uhlhaas P. J. (2012). Gamma-band activity in human prefrontal cortex codes for the number of relevant items maintained in working memory. *J. Neurosci.* 32 12411–12420. 10.1523/JNEUROSCI.0421-12.2012 22956832 PMC6621256

[B60] Rushworth M. F. S. Hadland K. A. Paus T. Sipila P. K. (2002). Role of the human medial frontal cortex in task switching: a combined fMRI and TMS study. *J. Neurophysiol.* 87 2577–2592. 10.1152/jn.00812.2001 11976394

[B61] Schaefer A. Gray J. R. (2007). A role for the human amygdala in higher cognition. *Rev. Neurosci.* 18 355–363. 10.1515/REVNEURO.2007.18.5.355 19544622

[B62] Schultze-Kraft M. Birman D. Rusconi M. Allefeld C. Görgen K. Dähne S. (2016). The point of no return in vetoing self-initiated movements. *Proc. Natl. Acad. Sci. U.S.A.* 113 1080–1108. 10.1073/pnas.1513569112 26668390 PMC4743787

[B63] Schwartenbeck P. FitzGerald T. H. B. Dolan R. (2016). Neural signals encoding shifts in beliefs. *Neuroimage* 125 578–586. 10.1016/j.neuroimage.2015.10.067 26520774 PMC4692512

[B64] Schwarz N. (2001). “Feelings as information. Implication for affective influences on information processing”, in *Theories of Mood and Cognition. A User‘s Guidebook* eds Martin L. L. Clore G. L. (London: Psychology Press) 159–176.

[B65] Schwarz N. (2011). “Feeling-as-Information theory”, in *Handbook of Theories of Social Psychology* eds Van Lange P. Kruglanski A. Higgins E. T. (London: Sage Publishing).

[B66] Seitz R. J. (2017). “Beliefs and believing as possible targets for neuroscientific research”, in *Process of Believing: The Acquisition, Maintenance, and Change in Creditions* eds Angel H.-F. Oviedo L. Paloutzian R. F. Runehov A. L. C. Seitz R. J. (Dordrecht: Springer) 69–80. 10.1007/978-3-319-50924-2_5

[B67] Seitz R. J. Angel H. F. (2014). Psychology of religion and spirituality: meaning making and processes of believing. *Relig. Brain Behav.* 5 118–178. 10.1080/2153599X.2014.891249

[B68] Seitz R. J. Franz M. Azari N. P. (2009). Value judgments and self-control of action: the role of the medial frontal cortex. *Brain Res. Rev.* 60 368–378. 10.1016/j.brainresrev.2009.02.003 19285106

[B69] Seitz R. J. Nickel J. Azari N. P. (2006). Functional modularity of the medial prefrontal cortex: involvement in human empathy. *Neuropsychology* 20 743–751. 10.1037/0894-4105.20.6.743 17100519

[B70] Seitz R. J. Paloutzian R. F. Angel H.-F. (2016). Processes of believing: Where do they come from? What are they good for? *F1000Research* 5:2573 10.12688/f1000research.9773.2 PMC5200943 28105309

[B71] Sensen M. Berg G. (2017). “Decision-making and credition under a microbial perspective”, in *Process of Believing: The Acquisition, Maintenance, and Change in Creditions* eds Angel H. F. Oviedo L. Paloutzian R. F. Runehov A. L. C. Seitz R. J. (Dordrecht: Springer) 443–450.

[B72] Sitaram R. Caria A. Veit R. Gaber T. Ruiz S. Birbaumer N. (2014). Volitional control of the anterior insula in criminal psychopaths using real-time fMRI neurofeedback: a pilot study. *Front. Behav. Neurosci.* 8:344 10.3389/fnbeh.2014.00344 PMC4196629 25352793

[B73] Stahl A. E. Feigenson L. (2015). Cognitive development. Observing the unexpected enhances infants‘ learning and exploration. *Science* 348 91–94. 10.1126/science.aaa3799 25838378 PMC5861377

[B74] Sugiura M. Seitz R. J. Angel H. F. (2015). Models and neural bases of the believing process. *J. Behav. Brain Sci.* 5 12–23. 10.4236/jbbs.2015.51002

[B75] Teske J. A. (2007). “Bindings of the will. The neuropsychology of subdoxastic faith”, in *Humanity, World and God – Understanding and Actions* eds Dress W. B. Meisinger H. Smedes T. A. (Lund: Lund University) 27–44.

[B76] Thorn C. A. Graybiel A. M. (2014). Differential entrainment and learning-related dynamics of spike and local field potential activity in the sensorimotor and associative striatum. *J. Neurosci.* 34 2845–2859. 10.1523/JNEUROSCI.1782-13.2014 24553926 PMC3931500

[B77] Toga A. W. Mazziotta J. C. (2000). *Brain Mapping. The Systems.* San Diego, CA: Acadamic Press.

[B78] Van Overwalle F. (2009). Social cognition and the brain: a metaanalysis. *Hum. Brain Mapp.* 30 829–858. 10.1002/hbm.20547 18381770 PMC6870808

[B79] Veit R. Konicar L. Klinzing J. G. Barth B. Yilmaz Ö. Birbaumer N. (2013). Deficient fear conditioning in psychopathy as a function of interpersonal and affective disturbances. *Front. Hum. Neurosci.* 7:706 10.3389/fnhum.2013.00706 PMC3829462 24298245

[B80] Wegner D. M. (2003). The mind’s best trick: how we experience conscious will. *Trends Cogn. Sci.* 7 65–69. 10.1016/S1364-6613(03)00002-0 12584024

[B81] Young G. B. Pigott S. E. (1999). Neurobiological basis of consciousness. *Arch. Neurol.* 56 153–157. 10.1001/archneur.56.2.153 10025420

