# Supplementary material for: Violations of Expectations As Matter for the Believing Process
Source: Front Psychol. 2017 May 29;8:772. doi: 10.3389/fpsyg.2017.00772 (PMC5446980; doi:10.3389/fpsyg.2017.00772)
Supplement: Supplementary file 1 [file Data_Sheet_1.DOCX]

**Box 1: Basic Terms**

When we change our focus from a static concept of belief to a concept of believing, the accent will be on the fluidity of those processes that merge internal and external reality and those that mediate their stable appearance. We cannot make this change within the boundaries of our past terminology and theoretical framework. Therefore, in the credition model it is necessary to introduce some new terms that make it possible to stress the process character of belief formation (Angel 2017).

**Bab:**

- The term *bab* was derived in analogy to the wooden toy babushka contains several figures of the same shape but different sizes
- The neologism “bab” means: “proposition including its emotional tinge”

**Bab and Process**

Babs are fluid basic units that transport content (i.e. an abstract proposition) in combination with the specific mightiness of emotional loadings”.

**Blob:**

Blobs are non-conscious babs

**bab-blob-configuration:**

Ensemble of different babs and blobs

**Box 2: Characteristics of a Single Bab:**

Emotions can be of the same type but differ in intensity. Therefore, we need to express the variabile intensity of emotional loadings of a bab. Propositionally identical babs can differ by their “weight” or “mightiness” of their emotional loadings. To express this dynamic fluidiy the terms *mega-bab* and *mini-bab* to signify the mightiness of an emotion were introduced. Within one person, the same propositional content may change from situation to situation and be “filled” or “coloured” with a different mightiness of emotional loadings. This fluid or dynamic character of the emotional mightiness of a bab might be called the babushka effect, an expression that may be found in different contexts but has lacked a clear scientific label until now (Angel 2017, 27).

**Box 3: Functional Units of the Believing Process**

The process of believing includes various mental operations that are heavily involved in the perception of events or objects in the external world and in the control of one’s behavior (Sugiura et al. 2015; Angel 2017):

- Enclosure function: affording perception
- converter function: affording (preparing) action
- Stabilizer function: affording learning and attitude
- Modulator function: related to individual biology
